# Optical coherence tomography-guided orbital atherectomy for calcified coronary lesions: rationale and design of the CROWN study

**DOI:** 10.1007/s12471-026-02055-5

**Published:** 2026-08-04

**Authors:** Eleni Ntantou, Alexandros A. Siskos, Nicolas M. van Mieghem, Joost Daemen, Isabella Kardys, Koen Teeuwen, Gioel G. Secco, Jan-Malte Sinning, Daniele Forlani, Wijnand K. den Dekker

**Affiliations:** 1 Department of Cardiology, Cardiovascular Institute, Thoraxcenter, Erasmus MC, University Medical Center Rotterdam, Rotterdam, The Netherlands; 2 Department of Cardiology, Catharina Hospital, Eindhoven, The Netherlands; 3 Interventional Cardiology and Cardiac Surgery Unit, SS Antonio e Biagio e Cesare Arrigo Hospital, Alessandria, Italy; 4 Department of Cardiology, St Vincent Hospital, Cologne, Germany; 5 Department of Cardiology, Santo Spirito Hospital, Pescara, Italy

**Keywords:** Calcific coronary disease, Orbital atherectomy system, Optical coherence tomography, Minimum stent area

## Abstract

**Background:**

The Orbital Atherectomy System (OAS) has demonstrated high procedural success, with excellent stent deliverability and low complication rates. The effects of orbital atherectomy (OA) and its impact on calcified coronary plaques are not yet fully understood, highlighting the need for a larger and more comprehensive study.

**Objective:**

Calcium Reduction by Orbital Atherectomy in Western Europe (CROWN) is an ongoing study that aims to evaluate the effects of OA in treating de novo, severely calcified coronary lesions before stent placement using optical coherence tomography (OCT), and to assess stent expansion by measuring the OCT-derived minimum stent area (MSA).

**Methods:**

The CROWN study is a prospective, multicenter, international, single-arm observational study. We will enroll 100 patients with severely calcified coronary lesions undergoing OCT-guided orbital atherectomy and stent placement. All patients will undergo peri-procedural OCT imaging, with assessments conducted before and after OA, as well as following stent placement. Patients will be enrolled at a maximum of 6 sites in the Netherlands, Germany, and Italy. The primary endpoint is to assess the proportion of patients achieving stent expansion, defined as an OCT-derived MSA ≥ 5.5 mm^2^.

**Conclusion:**

The CROWN study will evaluate the impact of OA on calcified plaques, potentially refine operator practices, and provide additional insights on device selection for treating severely calcified lesions (clinicaltrials.gov NCT06035783).

**Supplementary Information:**

The online version of this article (10.1007/s12471-026-02055-5) contains supplementary material, which is available to authorized users.

## Introduction

Calcific coronary artery disease is one of the most challenging pathologies in the interventional cardiology field. The presence of calcium prevents adequate stent placement and expansion. This can lead to accelerated neo-atherosclerosis and stent thrombosis [1]. The prevalence of calcific coronary artery disease is rising among patients with coronary artery disease, driven by an aging population and a higher incidence of comorbidities such as renal insufficiency, hypertension, and diabetes mellitus [2–4].

Although severely calcified lesions have an incidence of 6–20% among coronary artery disease patients, the amount of calcium is often underappreciated [2, 5, 6]. Mintz et al. reported low rates of detection and classification for coronary artery calcification (none/mild, moderate, and severe) with angiography (38%) but higher values (73%) with intravascular ultrasound (IVUS) [5].

The combination of low detection rates and underestimation of coronary calcified lesions by conventional angiography, along with the poor compliance of these lesions for stent placement without optimal preparation, contributes to elevated rates of major adverse cardiovascular events (MACE), increased cardiac mortality, and higher incidence of revascularization [1, 6–10].

Several techniques are available for modifying calcified lesions to facilitate optimal stent placement, including high-pressure and super-high-pressure non-compliant balloons, cutting or scoring balloons, and atherectomy devices.

Currently available atherectomy devices are rotational atherectomy (RA, Rotablator, Boston Scientific), laser atherectomy (ELCA, Philips), and orbital atherectomy system (OAS, Diamondback 360, Abbott).

Optimal preparation of severely calcified coronary lesions is of paramount importance. Intravascular imaging can confirm whether calcium was optimally prepared by showing fracturing of calcium (both superficial and medial) and/or superficial sanding. Adequate treatment of coronary calcification results in improved stent expansion and better clinical outcomes [11]. Over the past decade, post-percutaneous coronary intervention (PCI) physiology has gained prominence, with patients achieving a fractional flow reserve (FFR) > 0.90 after intervention experiencing significantly lower event rates [12]. It has been demonstrated that good stent expansion with a minimum stent area (MSA) > 5.5 mm^2^ is directly correlated with an FFR > 0.90, adding extra value to imaging-guided intervention [13].

The OAS demonstrated high procedural success, including high rates of successful stent deliverability and low rates of complications [2, 14–19]. The main feature of the device is the eccentrically mounted, diamond-coated crown that rotates at high speed within the coronary artery in an orbital motion, taking advantage of the centrifugal force created between the wire and the crown. This orbital motion of the crown can debulk severely calcified lesions by sanding superficial calcium and generating pulsatile forces that affect deep calcium [2, 16–19]. In the pivotal ORBIT trials, no imaging was performed, and large prospective studies using a stent optimization protocol to guide orbital atherectomy (OA) and core lab analysis with prespecified criteria are lacking [14, 15].

The recent randomized controlled ECLIPSE trial investigated whether vessel preparation with OA is superior to conventional balloon angioplasty in the treatment of severely calcified coronary artery lesions prior to implantation of contemporary drug-eluting stents (DES). The study found that routine use of OA before DES implantation did not result in a greater MSA or a reduced rate of target vessel failure (TVF) at one year, compared with a strategy based on balloon angioplasty alone. Importantly, the trial did not incorporate an optimization protocol based on prespecified intravascular imaging criteria, as imaging was only performed in a subset of patients [20].

We designed the CROWN study to further elucidate the effects of OA on coronary calcified plaques and MSA. Furthermore, by combining orbital atherectomy with intracoronary imaging, we aim to optimize the treatment of severely calcified lesions.

## Methods

### Study design

Calcium Reduction by Orbital Atherectomy in Western Europe (CROWN) is an investigator-initiated, prospective, multicenter, international, single-arm study. Patients with significant and severely calcified coronary lesions in need of advanced calcium modification (according to operator’s discretion) will undergo optical coherence tomography (OCT) guided OA and stent placement. OA will be performed in vessels with a reference diameter of at least 2.5 mm, in accordance with device instructions for use and contemporary practice. A total of 100 patients with calcified obstructive coronary artery disease eligible for OCT-guided PCI in need of advanced calcium modification will be enrolled. Ideally, all patients will undergo OCT before and after orbital atherectomy and after stent implantation, with at least 50 patients included at all three time points.

Written approval from the Ethics Committee (EC) with authority over the participating site was obtained prior to the start of the study. The study was conducted according to the Declaration of Helsinki, ISO 14155, and 21CFR Part 11 and received EC approval on 16 November 2022. ICH-GCP is used as guidance.

The first patient was enrolled in March 2024, and by June 2025, a total of 41 patients had been enrolled in the study. Patient enrollment is planned to continue until the end of the third quarter of 2026.

### Patient population

General inclusion and exclusion criteria are summarized in Tab. 1. Patients undergoing PCI of a severely calcified coronary lesion in need of advanced calcium modification to enable proper stent placement are being screened.

**Table 1:** Inclusion/exclusion criteria

Inclusion criteria	Exclusion criteria
De novo significant native coronary artery lesion	Left main disease
The target lesion must have evidence of severe calcification	Prior stenting of the target vessel
The target vessel reference diameter is ≥ 2.5 mm and ≤ 4.0 mm and the lesion does not exceed 40 mm in length	Target lesion has a thrombus or dissection
Age ≥ 18 years	Known LVEF ≤ 25%
The patient is an acceptable candidate for treatment with a drug-eluting stent in accordance with the applicable guidelines on percutaneous coronary interventions, manufacturer’s Instructions for Use and the Declaration of Helsinki	Diagnosed with chronic renal failure (GFR < 30 mL/min/1.73 m^2^)
Patient indication, lesion length and vessel diameter of the target lesion(s) are according to the ‘Instructions for Use’ that come with the Diamondback 360 ° Coronary OAS	Confirmed pregnancy
The patient is willing and able to cooperate with study procedures and required follow-up visits	Life expectancy < 12 months
The subject or legal representative has been informed of the nature of the study and agrees to its provisions and has provided an EC-approved written informed consent, including data privacy authorization	Age < 18 years
	Coronary anatomy that prevents delivery of OCT catheter
	Known allergy to soybean oil, egg yolk phospholipids, glycerin or sodium hydroxide

*EC* ethics committee; *GFR* glomerular filtration rate; *LVEF* left ventricular ejection fraction; *OAS* orbital atherectomy system; *OCT* optical coherence tomography

Definition of a severely calcified coronary lesion by angiography and OCT:

- Angiography: Presence of radiopacities noted without cardiac motion prior to contrast injection involving both sides of the arterial wall with calcification length of at least 15 mm and extending partially into the target lesion.
- Intravascular imaging (OCT): presence of ≥ 270° of calcium or lumen protruding calcified nodule(s) at > 1 cross-section.

This definition of severe calcification is consistent with that used in previous OCT-based studies, including the recently published ECLIPSE trial [20, 21].

### Primary endpoint

The main study endpoint is the proportion of patients who reach a stent expansion ≥ 5.5 mm^2^ as assessed by OCT (Tab. 2).

**Table 2:** Primary and secondary endpoints

**Primary endpoint:**
The proportion of patients who reach stent expansion ≥ 5.5 mm^2^ as assessed by OCT.
**Secondary endpoints:**
Clinical endpoints (measured at discharge, 30 days and 12 months):
- Procedural success, defined as successful stent delivery with:
- Final Core Lab defined TIMI flow of III
- Angiographic in-stent DS ≤ 20%
- Absence of in-hospital MACCE (consisting of all-cause death, spontaneous MI, TVR, stroke)
- TVF (cardiac death, target vessel spontaneous MI, and TVR)
- MACE (consisting of all-cause death, spontaneous MI, and repeat revascularization)
- Individual components of MACE and TVF
- Incidence of periprocedural MI:
- Type 4a (4th universal definition)
- Major angiographic intraprocedural complications including type C–F dissections, perforations, persistent slow flow or persistent no reflow, thrombus and major side branch occlusion (>2 mm)
- Probable and definite stent thrombosis
- Need for renal replacement at 30 days
**Intracoronary imaging endpoints (OCT) (Core Lab Assessed):**
- Final MSA
- Percentage of stent expansion
- Percentage of lumen area gain post OA and post stenting
- Minimal lumen area (MLA) post OA and post stenting
- Number of calcium fractures
- Number of calcium fractures based on calcium thickness post OA
- Incidence of OCT defined hematomas post OA
- Incidence and quantification of dissections post OA
- Number of calcified nodules modified post OA
**Angiographic endpoints (Core Lab Assessed):**
- Lesion length, diameter percentage of stenosis, reference vessel diameter
- In-stent and in-segment diameter stenosis
- In-stent and in-segment minimal lumen diameter (MLD)
- In-stent and in-segment acute gain

Abbreviations: *DS* diameter stenosis; *MACCE* major adverse cardiac and cerebrovascular events; *MACE* major adverse cardiovascular events; *MI* myocardial infarction; *MSA* minimum stent area; *OA* orbital atherectomy; *OCT* optical coherence tomography; *TIMI* thrombolysis in myocardial infarction; *TVF* target vessel failure; *TVR* target vessel revascularization.

### Secondary endpoints

The secondary endpoints are divided into three subgroups: clinical, intracoronary imaging, and angiographic endpoints (Tab. 2).

### Study procedures

**Fig. 1:**
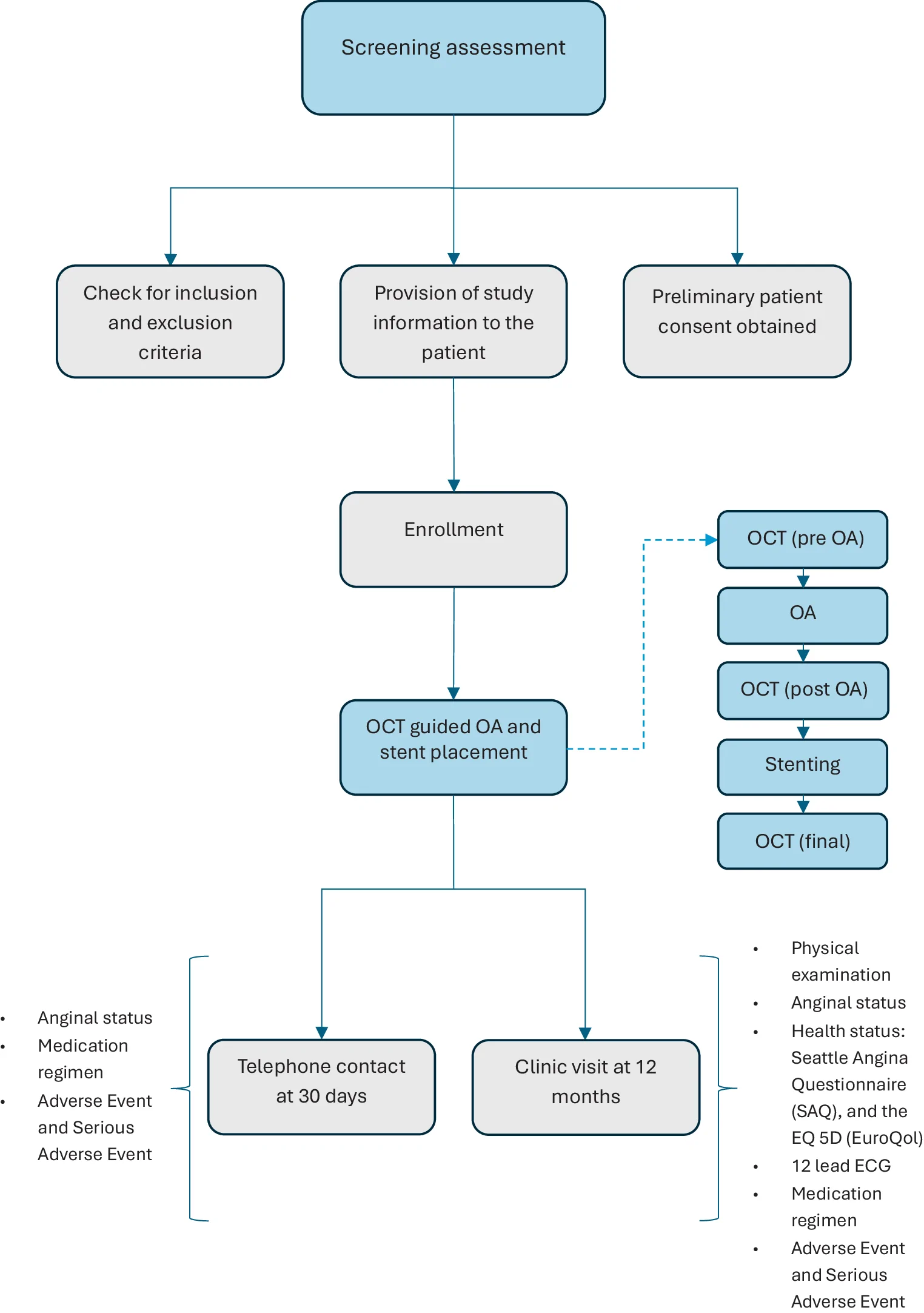
CROWN study Flow Chart. (*OCT* optical coherence tomography; *OA* orbital atherectomy)

Figure 1 depicts the flowchart of the study. Once severe coronary calcification has been confirmed and the patient has provided informed consent, the patient is considered enrolled in the study (see Supplementary Material). In brief, patients will undergo PCI following orbital atherectomy, with OCT performed before and after orbital atherectomy and again after PCI. A full description of study procedures—including OCT image acquisition, the OCT optimization protocol, stent delivery, angiography-based optimization, and post-stent OCT is provided in the Supplementary Material.

### Follow-up

Patients will be followed for up to one year after hospital discharge following the index procedure. This includes a telephone contact at 30 days and a clinic visit at 12 months to obtain information regarding cardiovascular drug use, hospitalizations, invasive and noninvasive diagnostic tests (ESM Table S1).

## Statistical consideration

### Sample size calculation

Patients with post-PCI FFR < 0.90 have an increased incidence of major adverse events, including repeat revascularization (20.3%), on the other hand, patients with post-PCI FFR > 0.90 have a significantly lower event rate (6.2%) [12, 22]. A threshold of stent expansion by OCT-derived MSA of 4.5 mm^2^ is discriminative for patients with MACE [23]. However, only a stent expansion by OCT-derived MSA ≥ 5.5 mm^2^ is correlated with an FFR > 0.90 [13]. A prospective randomized sub-study of the PREPARE-CALC trial using OCT, which randomized patients with severely calcified lesions treated with modified balloons (MB) or RA, had a primary endpoint of percentage of stent expansion as assessed by OCT. Results were neutral for the primary endpoint; however, in the context of secondary endpoints, absolute MSA was measured, and 82.5% and 79.4% from the MB and RA groups, respectively, reached an MSA ≥ 4.5 mm^2^ with a mean MSA of 6.1 ± 1.7 and 6.2 ± 1.9, respectively [24]. Considering these results and the absence of an optimization protocol in this sub-study, we assume that an OCT-derived MSA ≥ 5.5 mm^2^ can be achieved in at least 85% of the enrolled patients. The primary objective of this study is to determine the proportion of patients with stent expansion after the procedure, defined as OCT-derived MSA ≥ 5.5 mm^2^. With an expected proportion of 0.85, a confidence level of 95%, and a desired total width of the confidence interval of 0.15, we need to include 100 patients.

## Statistical analysis

### Primary study parameter(s)

All principal analyses will be performed with data from the time of the index procedure. The primary endpoint is descriptive. The proportion of patients with the primary endpoint, i.e., stent expansion as defined by OCT-derived MSA ≥ 5.5 mm^2^, and its 95% confidence interval will be calculated. The analysis of the secondary study parameters is described in the Electronic Supplementary Material [ESM].

### Data collection and management

Data are handled confidentially and encoded, and are incorporated in a study environment. The handling of personal data complies with the General Data Protection Regulation (GDPR; in Dutch: Algemene Verordening Gegevensbescherming, AVG). Data collected for each patient are recorded in a web-based electronic Case Report Form (eCRF).

### Site monitoring

Each site will be visited for monitoring twice, starting as soon as possible after the first patient has been enrolled.

### Endpoint adjudication process

An independent Clinical Events Committee of medical experts will review all serious adverse event data to adjudicate secondary endpoints.

### OCT acquisition and analysis

Quantitative and qualitative OCT analyses will be performed systematically for every 1 millimeter by the Erasmus University Medical Center academic corelab, using dedicated software (QCU-CMS, Leiden University Medical Center, LKEB, Division of Imaging Processing, v.4.69) according to a previously defined methodology.

## Discussion

The CROWN study will evaluate the effects of OA in treating de novo, severely calcified coronary lesions using OCT.

OA and OCT have become increasingly important in the optimization of PCI in cases of (severely) calcified coronary artery disease. OA offers a unique conceptual advantage over RA by utilizing a differential ablation mechanism that selectively modifies calcium while preserving softer tissue, thereby improving vessel compliance and facilitating optimal stent expansion. Meanwhile, OCT has emerged as a high-resolution imaging modality that enhances procedural precision by accurately assessing plaque morphology, guiding stent deployment, and detecting complications such as malapposition and edge dissections.

The safety and efficacy of the Diamondback 360 ° Coronary Orbital Atherectomy System have already been shown in the ORBIT I and II trials, showing low rates of angiographic complications and a procedural success rate of 89.6%, exceeding the predefined optimal performance goal of 82% [14, 15].

The recent randomized controlled ECLIPSE trial demonstrated that OA prior to DES implantation was not superior to conventional PCI without atherectomy in terms of clinical and imaging outcomes. One potential explanation for this finding could be that patients with severely calcified lesions, for whom the investigator deemed atherectomy clearly indicated, for example, balloon-uncrossable lesions, were not included in the trial. As a result, the trial predominantly excluded patients with severely calcified coronary lesions—those in whom OA could have provided the greatest benefit—and instead primarily included patients for whom standard balloon angioplasty may have been sufficient for lesion preparation [20].

In contrast, CROWN specifically includes patients in whom the operator considers conventional non-compliant balloon predilatation insufficiently effective based on severe angiographic or OCT-defined calcium. Consequently, CROWN also investigates the population excluded from ECLIPSE, providing complementary mechanistic and procedural data in the most complex lesion subset.

An observational study analyzing 18 patients with severely calcified coronary artery lesions utilized pre- and post-procedure OCT to assess the impact of OA, demonstrating its effectiveness and safety in ablating calcified tissue [25].

Similarly, a retrospective study of 58 patients examined plaque modification and stent expansion following OA, also using OCT. The findings revealed that OA successfully modified calcium, enabling post-stent calcium fracture even in cases of thick calcification. Furthermore, a greater extent of calcium modification, defined as a round, concave, polished lumen calcium surface, was associated with increased calcium fracture, ultimately enhancing stent expansion [18].

The CROWN study will expand on these findings by including 100 patients, incorporating pre-OA, post-OA, and post-stenting OCT imaging to provide a more comprehensive assessment of calcium fracture, superficial sanding, and stent expansion following OA. This larger sample size and additional imaging will offer deeper insights into the impact of OA on optimizing PCI outcomes in calcified lesions.

Importantly, CROWN is the first study to use a pre-specified OCT optimization protocol to precisely characterize the effects of OAS and its impact on calcified coronary plaques.

The findings of this study will help optimize results in OCT-guided interventions for severely calcified lesions modified with OA, elucidate the effect of OA on calcified plaques, potentially improve or change operator practice, and ultimately provide additional data on device choice to treat severely calcified lesions.

## Conclusion

In conclusion, the CROWN study is a prospective, multicenter, international, single-arm  observational registry among patients with severely calcified coronary lesions in need of advanced calcium modification who will undergo OCT-guided OA and stent placement. The comprehensive data collected before and after OA, as well as following PCI, will be used to evaluate the effects of OA in severely calcified lesions, including its ability to facilitate stent delivery and optimize stent expansion.

## Supplementary Information

ESM1: Supplementary material 1

ESM2: Supplementary material 2

## Data Availability

All data supporting the findings of this work are included in the article.
