# Supplementary material for: Optical coherence tomography-guided orbital atherectomy for calcified coronary lesions: rationale and design of the CROWN study
Source: Neth Heart J. 2026 Aug 4;34(9):299–305. doi: 10.1007/s12471-026-02055-5 (PMC13504061; doi:10.1007/s12471-026-02055-5)
Supplement: Supplementary file 1 [file 12471_2026_2055_MOESM1_ESM.docx]

**Supplementary Information**

**Informed Consent**

All patients must give informed consent prior to enrolment in the study. In the event that the patient is unable to provide written informed consent because of acute setting, an initial verbal consent from the patient could be obtained. If a patient is providing verbal consent, an impartial witness must be present during the entire informed consent discussion. Where a patient has initially verbally consented, written consent should be sought from the patient as soon as the patient is capable of signing the consent form.

**Study procedures**

Before using the orbital atherectomy system (OAS), imaging with optical coherence tomography (OCT) is performed over a workhorse guidewire. After imaging, the workhorse guidewire is exchanged with the use of an over the wire (OTW) balloon or microcatheter for the ViperFlex wire, or the vessel is directly wired with the ViperFlex wire. The orbital atherectomy device (OAD) is advanced over the wire proximal to the lesion. Orbital atherectomy (OA) is performed by slowly advancing or retracting the 1.25mm crown through the calcified lesion, approximately 1mm per second. In this way circumferential calcium is sanded while deeper medial calcium can be fractured. The use of either 80.000 rpm or 120.000 rpm is left at the operators discretion, with a protocol defined preference to use 80.000 rpm. After satisfactory plaque modification, the OAD is removed and the ViperFlex wire will be exchanged for a workhorse guidewire with the use of an OTW balloon or a microcatheter. During atherectomy, activated clotting time (ACT) is kept above 250 seconds and use of atropine is at the operator’s discretion. After OA, imaging with OCT is repeated. Balloon dilatation before the use of the OAS is not advocated to prevent occurrence of dissections, but after OA treatment balloon dilatation can be performed before and after stent placement. After stent placement final imaging with OCT is performed. Optimal stent placement according to pre-specified criteria is assessed and when necessary additional treatment is performed. Antithrombotic therapy after procedure is prescribed according to the current guidelines and focused updates [1,2].

**Optical coherence tomography image acquisition**

The commercially available system OPTIS Integrated System and Mobile System with OPTIS Software in combination with Dragonfly Optis, Dragonfly OpStar or Optis DUO imaging catheters (Abbott vascular, Santa Clara, California) is used to acquire OCT images.

The catheter is advanced until at least 5 mm more distal of the distal edge of the target lesion and by use of the integrated automated pull-back device at 36 mm/s, the length of the region of interest is scanned.

A 75mm pullback in 2.1 seconds with 5 frames per mm is recommended. Contrast injection volume with power injectors (ACIST or Medrad) is recommended to allow sufficient contrast clearing. During OCT pullback it is recommended to perform cine angiography in order to maximize utility of delivered contrast.

**OCT optimization protocol**

OCT will be used prior to the use of OA. Predilatation with a balloon diameter of less than 2 mm is permitted before OCT to facilitate catheter passage. In case the Dragonfly Optis, Dragonfly OpStar or Optis DUO imaging catheters (Abbott vascular, Santa Clara, California), despite predilatation with a small diameter balloon, is not able to pass through the lesion, the patient cannot be included into the study. Post-OA OCT will be repeated and the reference sites will be determined.

**Reference segments:** The optimization procedure requires the choice of 2 reference segments. Those segments are to be located at the proximal and distal edge of the target lesion. Reference segments extend at least 5 mm length beyond the planned stent edges. Edges should meet the following criteria: absence of lipidic plaques, coverage of diseased areas with an aim for reconstruction of lumen at the lesion using the lumen profile tool, coverage of dissections inflicted by predilatation, coverage of plaque ruptures adjacent to the intended stent landing zone, coverage of areas with cross-sectional area (CA) < 4.5 mm^2^ [3].

**Choice of stents:**

- Stent diameter: Choice of diameter is based on the OCT measurement of mean external elastic lamina (EEL) diameter at distal reference site if visible, the diameter is rounded-down to the next stent available unless the mean EEL diameter is equal to an existing stent size, then no round-down is required. The mean diameter of multiple EEL diameter measurements is the primary determinant for selecting the stent diameter. However, if imaging does not allow for multiple measurements and only a single EEL diameter is available, then that measurement will be used to determine the stent diameter. In the case of non-visible EEL, the mean lumen diameter of the distal reference segment guides the choice of the stent and a round-up to the next available stent size is made even if the measured diameter is the same with an existing stent size.
- Stent length: Reference vessel markers are to be added to both proximal and distal lesion edges. Segments with visible media have to be present at both sites within 5 mm and at least >180°. Once both proximal and distal lumen borders of the reference segments can be measured then this reference cross-section is selected and the stent length is determined using the OCT Lumen Profile Software.

**Stent delivery and angio-based optimization:**

Stent implantation is advised to be guided by angiographic co-registration if available. Angiographic assessment is made after initial stent implantation and post dilation with non-compliant balloon can be performed. Choice of balloons diameter should not be larger than the closest post-OA OCT mean reference vessel EEL or 0.5mm larger if the EEL was not measurable.

**OCT after stent delivery**

Once stenting of the target vessel is completed and the angiographic result is deemed optimal and all interventional equipment would be otherwise removed, OCT is repeated for stent expansion assessment. Stent is divided in two segments, first half is the proximal half, and the second half is the distal half. Proximal half stent segment is optimized with the MLA (minimum lumen area) of the proximal reference segment and the distal half stent segment is optimized with the MLA of the distal reference segment. An optimal stent expansion is defined as a minimum stent area (MSA) of ≥85% of the corresponding reference lumen. In case of stent underexpansion (criteria for stent expansion not met) post-dilatation with non-compliant balloons should be performed with the same balloon criteria choice of angiographic assessment post-dilation.

After post dilation and OCT-guided optimization of stent expansion, proximal and distal reference edges, defined as 5 mm from the edges of the stent, are examined for edge dissection or residual plaque. A major edge dissection (≥ 3mm in length and ≥60 degrees of the circumference of the vessel at site of dissection) also requires treatment with additional stent placement unless anatomically prohibitive.

Major stent malapposition also needs to be treated with the same balloon criteria as angiography assessment post-dilation. There is major malapposition when the distance between strut and vessel wall is >350µm or, <350µm and >200µm for a length >6mm [4].
In the presence of major lipid or calcified plaques at the stent edge landing zone, the stent should be extended into more healthy-appearing segments. The stent should also cover any ruptured plaques within the intended landing zone. While it is acceptable to stent short healthy vessel segments if needed to ensure complete lesion coverage, efforts should be made to avoid covering adjacent side branches whenever possible. A CA <4.5mm² at either proximal or distal reference segments requires treatment with an additional stent.

In case of long-stenting (stent’s length ≥28mm), if the proximal and distal reference lumen dimensions are different by ≥0.5mm, then multiple non-compliant balloons of different diameters should be used to achieve optimal stent expansion of each segment. If the underexpansion is located in the mid segment of a long stent (≥28mm), the choice of the balloon for post-dilation is based on the average of the proximal and distal reference measurements.

The protocol is completed with a final OCT where MSA is assessed. If further percutaneous coronary intervention (PCI) is needed based on findings from the post-PCI OCT (in accordance with optimization protocol) an additional OCT run must be conducted.

**Statistical analysis**

**Secondary study parameter(s)**

Distribution of continuous variables will be tested for normality using the Kolmogorov Smirnov test. The secondary parameters will be described as follows, according to their distributions:

- Categorical variables will be described as numbers and percentages.
- Continuous variables with normal distributions will be described as means and standard deviations.
- Continuous variables with non-normal distributions will be described as medians with 25th-75th percentile.
- Changes between time points will be calculated as continuous variables and described as means and standard deviations (normal distributions) or as medians with 25th-75th percentile (non-normal distributions).

To obtain the estimates of major adverse cardiovascular events (MACE) and **target vessel failure (**TVF) at 30 days and 1 year post intervention, we will apply the method of Kaplan-Meier.

**References**

1. Neumann FJ, Sousa-Uva M, Ahlsson A, et al. 2018 ESC/EACTS guidelines on myocardial revascularization. EuroIntervention. 2019;14:1435–534.
2. Valgimigli M, Bueno H, Byrne RA, et al. 2017 ESC focused update on dual antiplatelet therapy in coronary artery disease developed in collaboration with EACTS. Eur J Cardiothorac Surg. 2018;53:34–78.
3. Holm NR, Andreasen LN, Neghabat O, et al. OCT or angiography guidance for PCI in complex bifurcation lesions. N Engl J Med. 2023;389:1477–87.
4. Taniwaki M, Radu MD, Zaugg S, et al. Mechanisms of very late drug-eluting stent thrombosis assessed by optical coherence tomography. Circulation. 2016;133:650–60.
