# Supplementary material for: Optical coherence tomography-guided orbital atherectomy for calcified coronary lesions: rationale and design of the CROWN study
Source: Neth Heart J. 2026 Aug 4;34(9):299–305. doi: 10.1007/s12471-026-02055-5 (PMC13504061; doi:10.1007/s12471-026-02055-5)
Supplement: Supplementary file 2 [file 12471_2026_2055_MOESM2_ESM.docx]

| Supplementary Table S1. Follow-up planning | | | | | |
| --- | --- | --- | --- | --- | --- |
| Event | Screen | Procedure | Post - Procedure to Hospital discharge | 30 Days | 12 Months |
| Type of contact |  |  |  | Telephone contact | Outpatient clinic visit |
| Inclusion/ exclusion Criteria | X |  |  |  |  |
| Informed consent | X |  |  |  |  |
| Physical examination | X |  |  |  | X |
| Medical and Cardiac history | X |  |  |  |  |
| Anginal Status | X |  | X | X | X |
| Health status: Seattle Angina Questionnaire (SAQ), and the EQ 5D (EuroQol) |  |  |  |  | X |
| CBC, blood chemistry, lipid panel, liver function tests^1^ | X^1^ |  |  |  |  |
| CK, CK-MB | X^2^ |  | X^3^ |  |  |
| Troponin and renal function | X |  | X |  |  |
| 12 lead ECG | X |  | X^4^ |  | X |
| Medication regimen^5^ | X | X | X | X | X |
| Adverse event and Serious Adverse Event* |  | X | X | X | X |
| Angiography (QCA) |  | X |  |  |  |

CBC, complete blood count; CK, creatine kinase; CK-MB, creatine kinase–myocardial band; ECG, electrocardiogram; QCA, quantitative coronary angiography;

^1^ Blood test suggested prior to procedure

^2^ CK, CK-MB are suggested to be drawn within 2 hours prior to PCI

^3^ CK, CK-MB are suggested to be determined 6, 12 and 18 hours after PCI or at discharge. At least 2 measurements are suggested to be done between 8 and 24 hours, with at least 4 hours time difference between the measurements

^4^ Suggested within 24 hours post-procedure or at discharge, whichever comes first

^5^ All concomitant cardiovascular medication should be recorded starting 24 hours prior to index procedure through the 12 month follow up period.

* Up to 12 month follow up all adverse events need to be collected.
